# Impact of Tai Chi and Aerobic Exercise on Cognitive Function, Balance, Cardiovascular Fitness, and Quality of Life in Older Adults: Randomized Control Trial

**DOI:** 10.7759/cureus.62497

**Published:** 2024-06-16

**Authors:** Reema Joshi, Neha Kulkarni, Chaitanya A Kulkarni, Prachi Bansal

**Affiliations:** 1 Physiotherapy, Dr. D. Y. Patil College of Physiotherapy, Pune, IND; 2 Community Based Rehabilitation, Dr. D. Y. Patil College of Physiotherapy, Pune, IND; 3 Public Health, Datta Meghe Institute of Higher Education and Research, Wardha, IND; 4 Community Health Physiotherapy, Ravi Nair Physiotherapy College, Wardha, IND

**Keywords:** original article, fall prevention, geriatrics, cognition, chen tai chi

## Abstract

Introduction

Tai chi, an ancient Chinese martial art, was originally developed for combat and self-defense. Over time, it has evolved into both a sport and a form of exercise. This gentle, low-impact exercise involves performing a series of deliberate, flowing motions while focusing on deep, slow breaths. This study investigates the effects of chen tai chi and aerobic exercises on cognition, balance, cardiopulmonary fitness, and quality of life in older adults.

Methodology

This study employed a single-blinded randomized control trial design, enrolling 60 participants aged between 60 and 75 years. Participants were divided into three groups: Group A (aerobics), Group B (chen tai chi), and Group C (control). Exercise sessions were held four days per week over a period of four weeks. Evaluations included the Montreal Cognitive Assessment for cognition, a one-leg stand test for static balance, a Timed Up and Go Test for dynamic balance, a six-minute walk test for cardiopulmonary fitness, and a health-related quality-of-life scale. Assessments were conducted at baseline, immediately after the intervention, and at follow-up was taken after eight weeks.

Results

Post-intervention, improvements were observed in cognition and static balance across all groups. Within-group analysis revealed that the aerobics group experienced statistically significant enhancements in cognition (p = 0.0001) and static balance (p = 0.01). Although no statistically significant differences were found between groups in terms of dynamic balance, cardiopulmonary fitness, and quality of life, the within-group analysis showed significant improvements in the aerobics group in dynamic balance (p = 0.0009), cardiopulmonary fitness (p = 0.03), and quality of life (p = 0.0001).

Conclusion

Compared to chen tai chi and no intervention, the study concludes that aerobic exercise has a more pronounced effect on improving cognition, balance, cardiopulmonary fitness, and quality of life in older adults. Aerobic exercise is recommended as an effective method to prevent frailty and promote independence among the elderly.

## Introduction

A World Health Organization report from October 2022 indicates that people are experiencing longer lifespans globally, with a growing number expected to live into their 60s and beyond. By 2030, it is projected that one in every six individuals on earth will be aged 60 or older. The global population of those over 60 is estimated to increase from 1 billion in 2020 to 1.4 billion by 2030. In India, the proportion of individuals aged 60 and above has risen to 10.1% in 2021. It is anticipated to increase to 13.1% by 2031, according to the population census and report of the technical group on population projections for India and states 2011-2036 [1]. Another important factor is the gender inequalities in chronic diseases among older individuals in India. It was somewhat lower for men at 8.2%, while it was higher for women at 9.0%, as per the most current National Family Health Survey (NFHS) results [2].

The increase in systemic inflammation associated with aging, known as "inflame-aging," is a significant factor contributing to poor health in older individuals. This condition is characterized by elevated levels of pro-inflammatory markers, such as interleukin 6, in both the bloodstream and tissues, which are closely linked with aging. Various factors contribute to inflame-aging, including genetic predisposition, central obesity, heightened gut permeability, alterations in the microbiota, cellular senescence, activation of the NLRP3 inflammasome, oxidative stress from dysfunctional mitochondria, immune cell dysregulation, and persistent infections. Inflame-aging is believed to contribute to the development of multiple health conditions and increased frailty while also impacting body composition by causing sarcopenia (muscle loss) and an increase in fat mass. Aerobic exercise programs are beneficial for older individuals in maintaining or restoring functional independence and potentially preventing, delaying, or reversing frailty. Aerobic exercise involves repetitive and structured movements that require the body's metabolic system to utilize oxygen to produce energy. Research suggests that physical activity, particularly aerobic exercise, may improve cognitive function and counteract age-related declines in the brain's ability to adapt to external demands by enhancing neurogenesis and brain plasticity. The muscle groups targeted by aerobic exercise rely on aerobic metabolism, which utilizes oxygen to extract energy from amino acids, carbohydrates, and fatty acids to produce adenosine triphosphate (ATP). Tai chi, a traditional Chinese exercise with a history of over 3,000 years, promotes health and well-being in older adults. Chen-style tai chi, characterized by more complex choreography and skipping movements, may present greater cognitive function and balance challenges due to its intricate nature. For example, the continuous coordination of body movements while shifting weight from foot to foot requires coordination and balance. Modified tai chi, featuring more intricate movement patterns than tai chi-24, may be more effective in enhancing neural plasticity and crucial neurotrophins derived from the brain, which is particularly beneficial for elderly individuals [3]. It is, therefore, essential to draw attention to the various affections that influence cognition, balance, cardiopulmonary fitness, and quality of life. To make an older person independent to the point that he can perform his daily activities and have an improved quality of life without any obstacles, the present study will investigate the effectiveness of interventions like chen tai chi and aerobics exercises on any of these domains compared to a control group.

## Materials and methods

A randomized control trial, which included community-dwelling elderly of age between 60 and 75 years, was conducted for four weeks under the banner of an NGO in Pune. The ethical approval was obtained from the Institutional Review Board with the number DYPCPT/IEC/24/2022. The trial was also registered with the clinical trial registry India with CTRI/2022/10/046272. The screening of participants was done, and participants who had any recent serious injury, systolic blood pressure (BP) of more than 200 mmHg and diastolic BP of more than 120mm Hg, acute episode of cerebrovascular accident, severe cardiopulmonary conditions, recent fractures, grade 3 or 4 osteoarthritis (OA) knee and occurrence of recurrent falls were excluded from the study. Subjects were informed about the study protocol and purpose of the research, and those who were willing to participate were included according to the Declaration of Helsinki. Written informed consent was taken from all the participants.

Study participants

Participants were randomly assigned to one of group A (aerobics), group B (chen tai chi), and group C (control) - using simple random sampling via the chit method. Assessments were carried out at the start and the end of the eight weeks. Cognitive function was evaluated using the Montreal Cognitive Assessment (MOCA), Timed Up and Go Test for dynamic balance; physical function was measured using two tests: the Functional Reach Test, with a completion criterion of less than 6 inches, and the six-minute walk test, with a minimum distance criterion of 630 m. Follow-up assessments were conducted one week post-intervention.

Intervention

The duration of the intervention was a total of four weeks. During this, the respective participants were trained in order of their groups: chen tai chi, aerobics exercises, or control group with conventional exercises. In the first two weeks, groups A and B followed a protocol of 30 minutes with five minutes of warm-up, 20 minutes of respective exercises, and five minutes of cool-down. In the next two weeks, groups A and B followed the intervention, which was 40 minutes, with five minutes of warm-up, 30 minutes of exercises, and five minutes of cool-down. Groups A and B participants were accompanied throughout the exercise/test to ensure safety. The exercise/tests were stopped at a time during the procedure, keeping in mind the termination criteria. The assessment was taken pre-intervention, post-intervention, and as a follow-up one week after the completion of the intervention.

Statistical analysis

For data analysis, IBM SPSS Statistics 26.0 (SPSS Inc., Chicago, IL) was used. Descriptive statistics provided insights into the mean and standard deviation for each group. The Shapiro-Wilk test was conducted to check for normality in the data distribution it was passed normality checked. For non-parametric distributions, differences between two groups were analyzed using the Mann-Whitney U test and between more than two groups using the Kruskal-Wallis test. All statistical tests were performed with a significance threshold set at p < 0.05.

## Results

Table 1 shows the mean with a standard deviation of age, height, weight, BMI, and heart rate in the aerobics, tai chi, and control groups.

**Table 1:** Baseline demographic data

Characteristics	Aerobics (mean ± SD)	Tai chi (mean ± SD)	Control (mean ± SD)	p-Value
Age	66.7 ± 4.33	66 ± 5	66.4 ± 4.09	0.76
Height	1.58 ± 0.06	1.56 ± 0.06	1.59 ± 0.08	0.85
Weight	72.8 ± 8.39	66.39 ± 9.74	64.79 ± 10.62	0.06
BMI	29.09 ± 3.31	27.13 ± 3.39	26.50 ± 4.06	0.23
Heart rate	80.1 ± 6.64	78.05 ± 6.65	80.2 ± 7.92	0.55

The one-way ANOVA and p-value within the groups were done using repeated measures of ANOVA. The one-way ANOVA reported statistically significant differences between the groups for all except for tai chi with a control group and follow-up. Within-group analysis by repeated measures ANOVA test also reported a statistically significant difference in the aerobics group (Table 2).

**Table 2:** Within-group and between-group comparisons of cognition using MOCA MOCA - Montreal Cognitive Assessment

	Group	Aerobics (mean ± SD)	Tai chi (mean ± SD)	Control (mean ± SD)	p-Value (one-way ANOVA)	p-Value (post hoc)	
Cognition (MOCA)	Pre-intervention (A)	28.4 ± 1.11	28.45 ± 1.47	28.7 ± 1.14	0.75	1 vs. 2	0.86
						1 vs. 3	0.77
						2 vs.3	0.63
	Post-intervention (B); eight weeks	30.55 ± 0.67	29.25 ± 1.18	29.4 ± 0.8	0.0001	1 vs. 2	0.0001
						1 vs. 3	0.0001
						2 vs. 3	0.88
	Follow-up (C); one week	31.23 ± 0.67	29.35 ± 1.11	29.45 ± 0.67	0.0001	1 vs. 2	0.0001
						1 vs. 3	0.0001
						2 vs. 3	0.87
p-Value (repeated measures of ANOVA)		0.0001	0.69	0.72	-	-	
Post hoc test	A vs. B	0.0001	0.62	0.70	-	-	
	A vs. C	0.0001	0.71	0.69	-	-	
	B vs. C	0.01	0.94	0.91	-	-	

The one-way ANOVA and the p-value within the groups were done using repeated measures of ANOVA. The one-way ANOVA reported statistically significant differences between the groups for all except for tai chi with a control group and follow-up. Within-group analysis by repeated measures ANOVA test also reported statistically significant differences in the aerobics group (Table 3).

**Table 3:** Within-group and between-group comparison of static balance using one-leg standing test

	Group	Aerobics (mean ± SD)	Tai chi (mean ± SD)	Control (mean ± SD)	p-Value (Kruskal-Wallis test)	p-Value (post hoc)	
One-leg standing	Pre-intervention (A)	22 ± 10.42	20.85 ± 10.48	17.6 ± 7.39	0.29	1 vs. 2	0.91
						1 vs. 3	0.28
						2 vs. 3	0.50
	Post-intervention (B) eight weeks	29.95 ± 9.44	24.5 ± 11.21	19.45 ± 7.48	0.002	1 vs. 2	0.15
						1 vs. 3	0.001
						2 vs. 3	0.19
	Follow-up (C) one week	30.25 ± 10.09	24.55 ± 9.87	20.2 ± 7.61	0.002	1 vs. 2	0.10
						1 vs. 3	0.001
						2 vs. 3	0.27
p-Value (Friedman test)		0.01	0.41	0.48	-	-	
Post hoc test	A vs. B	0.04	0.48	0.69	-	-	
	A vs. C	0.02	0.47	0.47	-	-	
	B vs. C	0.96	0.78	0.93	-	-	

The p-value using a post hoc test between the groups is also given. The Kruskal-Wallis test reported a statistically significant difference between the groups at post-intervention and follow-up. Within-group analysis by the Friedman test also reported statistically significant differences in the aerobics group (Table 4).

**Table 4:** Within-group and between-group comparison of dynamic balance using Timed Up and Go Test

	Group	Aerobics (mean ± SD) (1)	Tai chi (mean ± SD) (2)	Control (mean ± SD) (3)	p-Value (Kruskal-Wallis test)	p-Value (post hoc)	
Timed Up and Go Test	Pre-intervention (A)	15.35 ± 3.97	14.75 ± 2.69	14.4 ± 2.06	0.59	1 vs. 2	0.79
						1 vs. 3	0.57
						2 vs. 3	0.92
	Post-intervention (B); eight weeks	13.2 ± 2.54	13.6 ± 2.13	13.2 ± 2.07	0.92	1 vs. 2	0.89
						1 vs. 3	0.91
						2 vs. 3	0.90
	Follow-up (C); one week	12.4 ± 3.13	13.15 ± 2.84	13.1 ± 1.98	0.78	1 vs. 2	0.83
						1 vs. 3	0.92
						2 vs. 3	0.79
p-Value (Friedman test)		0.0009	0.07	0.74	-	-	-
Post hoc test	A vs. B	0.09	0.23	0.14	-	-	-
	A vs. C	0.007	0.06	0.63	-	-	-
	B vs. C	0.98	0.79	0.42	-	-	-

The Kruskal-Wallis test did not report a statistically significant difference between the groups. However, within-group analysis by the Friedman test reported statistically significant differences in the aerobics group (Table 5).

**Table 5:** Within-group and between-group comparison of cardiopulmonary fitness using a six-minute walk test SMWT - six-minute walk test

	Group	Aerobics (mean ± SD)	Tai chi (mean ± SD)	Control (mean ± SD)	p-Value (Kruskal-Wallis test)	p-Value (post hoc)	
SMWT	Pre-intervention (A)	15.96 ± 1.84	16.11 ± 2.39	15.59 ± 2.54	0.74	1 vs. 2	0.97
						1 vs. 3	0.85
						2 vs. 3	0.74
	Post-intervention (B); eight weeks	17.14 ± 1.69	16.94 ± 2.52	16.25 ± 2.73	0.44	1 vs. 2	0.95
						1 vs. 3	0.44
						2 vs. 3	0.61
	Follow-up (C); one week	17.34 ± 1.85	16.84 ± 2.92	16.44 ± 3.09	0.55	1 vs. 2	0.81
						1 vs. 3	0.52
						2 vs. 3	0.88
p-Value (Friedman test)		0.03	0.45	0.59	-	-	-
Post hoc test	A vs. B	0.08	0.57	0.73	-	-	-
	A vs. C	0.03	0.48	0.60	-	-	-
	B vs. C	0.92	0.87	0.97	-	-	-

The Kruskal-Wallis test did not report a statistically significant difference between the groups. Within-group analysis by the Friedman test reported statistically significant differences in the aerobics group (Table 6).

**Table 6:** Within-group and between-group comparison of quality of life using health-related quality of life score HRQOL - health-related quality of life

	Group	Aerobics (mean ± SD)	Tai chi (mean ± SD)	Control (mean ± SD)	p-Value (one-way ANOVA)	p-Value (post hoc)	
HRQOL	Pre-intervention (A)	93.2 ± 1.11	93.95 ± 3.94	94.75 ± 3.25	0.24	1 vs. 2	0.88
						1 vs. 3	0.57
						2 vs. 3	0.73
	Post-intervention (B); eight weeks	95.5 ± 2.51	95.5 ± 3.24	96.15 ± 2.92	0.64	1 vs. 2	0.48
						1 vs. 3	0.53
						2 vs.3	0.46
	Follow-up (C); one week	96.05 ± 2.27	96.15 ± 2.78	96.7 ± 2.74	0.77	1 vs. 2	0.92
						1 vs. 3	0.84
						2 vs. 3	0.89
p-Value (repeated ANOVA)		0.0001	0.10	0.10	-	-	-
Post hoc test	A vs. B	0.002	0.31	0.29	-	-	-
	A vs. C	0.0001	0.10	0.09	-	-	-
	B vs. C	0.67	0.81	0.82	-	-	-

The one-way ANOVA test did not report statistically significant differences between the groups. Within-group analysis by repeated measures ANOVA reported a statistically significant difference in the aerobics group.

## Discussion

This study aimed to compare the effect of chen tai chi and aerobics exercise on cognition, balance, cardiopulmonary fitness, and quality of life in older adults. In this present study, 60 elderly individuals between the ages of 60 and 75 years were included, both male and female, with average BMI (kg/m^2^) and average maximum heart rate (beats per minute) [1]. In the current study, aerobics exercises specifically improved cognition more than tai chi and the control group. This suggests that increasing physical activity, particularly at mild to moderate intensity and involving frequent, interconnected movements and choreography, such as aerobics, is beneficial. The participant begins comprehending, grasping, and performing the movement in the first two to three sessions. Later, they begin to replicate and imitate the movement pattern and recall previously performed movements from their long-term memory, which engages the hippocampus, amygdala, and neocortex and helps to sustain and store the movement pattern over an extended period. Aerobics challenged the cognitive domains, particularly attention, memory, perceptual-motor control, and executive function. This study shows that aerobic exercise aids in maintaining the health of the white matter tracts that lead to and from the prefrontal areas [2]. Static balance using one-leg standing and dynamic balance using the Timed Up and Go Test were found to be significantly improved by aerobic exercises because aerobic exercises require participants to continuously change their body direction while shifting from a symmetrical to an asymmetrical base of support; they were found to significantly improve static balance while standing on one leg. This is because aerobic exercises encourage more single-leg stance shifts, weight-bearing capacity, and balance. Numerous dimensions of aerobic exercise are also considered, such as changes in the center of mass (COM) in vertical and horizontal axes, which especially challenge participants [3]. With these COM changes, the dual tasking involved in aerobics activities probably stimulates sensory and neuromuscular control systems, which helps older people with postural control. When engaging in aerobic exercise, lifting the body weight fast and repeatedly requires significant muscle power; it is therefore hypothesized that as muscle power rises, so does postural stability. Therefore, increasing muscular power should result in increasing balance improvement [4]. The multimodal sensory system gives information about the body's position as it moves across the ground when engaging in this type of workout training. Numerous sensory receptors in the eyes (visual input), inner ears (vestibular input), and skin (kinesthetic input) provide feedback to the central nervous system so that the appropriate muscles can be activated for the movement to occur. The memory retrieval, planning, and executive function to enact a particular movement is also improved in this type of exercise [5]. Cardiopulmonary fitness, as assessed by the six-minute walk test, also shows a statistically significant difference in the aerobics group. Aerobics is a holistic approach that trains the entire cardiopulmonary system to efficiently respond to moderate physical stress induced by exercise. The buoyant music and rhythmic movements additionally reduce overall physical and mental fatigue, encouraging participants to exercise for longer durations and engage more zealously in daily activities compared to before [6]. In the aerobic exercise group, there was a statistically significant trend toward improved aerobic capacity as well as an increase in the number of hours spent engaging in moderate functional activity, the amount of energy expended each week, and other indicators of increases in aerobic physical activity. According to a study by Raul A. Martins published in 2010, aerobic training was found to reduce total cholesterol (TC), low-density lipoprotein cholesterol (LDL-C), and triglycerides (TG) while increasing HDL-C and increase six-minute walk distance by 13% in sedentary older adults [7]. Aerobic exercises have also shown statistically significant differences in quality of life using the Health-Related Quality of Life Score (HRQL). As previously mentioned, when the body's physical and mental fatigue is reduced, functional activity and energy expenditure automatically increase. Shumaker and colleagues examined the impact of aerobic exercise on the quality of life of 90 urinary incontinent women. They found that aerobic exercise improved physical functioning, emotional well-being, social functioning, mental health status, role activities, health perceptions, and overall life satisfaction. [8]. Tai chi exercises have shown improvement in cognition using MOCA, as more complex movement patterns may be more effective in enhancing neural plasticity because when performing a movement repeatedly over time, the brain’s ability to restructure and rewire itself is also improved, and its adaptability also increases. Tai chi exercises also help to improve the key brain-derived neurotrophic molecules, which also maintain high expression and regulation of both excitatory and inhibitory synaptic transmission, which is also increased by improving the learning and memory capacity of the cognitive domains. Tai chi has also shown improvement in static balance using one-leg standing and dynamic balance using the Timed Up and Go Test. This may be because tai chi, although a neuromuscular balance and coordination exercise, begins with the wuji (void) stance, which lowers the body and creates a lower center of gravity. This stance increases the base of support, imparting more stability. Since the exercise initiates from a more stable position and is maintained throughout, the weight shifting occurs at a slower rate and pace. Consequently, the destabilizing forces a participant encounters to promote proactive and reactive balance strategies are not as challenging compared to other exercise forms. Additionally, tai chi's semi-squat posture, which puts strain on the knee muscles, has an impact on training. The amplitude of weight shifting, as determined by the degree of COP displacements, varies among different tai chi motions and from person to person. Therefore, the COM alterations that occurred during aerobic activity were superior and may account for the differences between these two groups that we found in our study [9]. Because tai chi is a slow-paced neuromuscular balance and coordination test, it did not adequately challenge the cardiopulmonary system, and there were no changes in maximal aerobic, and as a result, the six-minute walk distance did not increase [10]. Also, it did not change the mood or encourage participants to participate more actively. Therefore, the quality of life was not affected as much as aerobics [11]. The control group was advised to perform certain exercises at home. It was noted that the control showed no significant changes as compared to aerobics and tai chi because the participants in this group were not as regular and disciplined as the other group participants [12]. They could not manage to make time for exercises and inculcate in their daily routine by themselves. The awareness among people of this group regarding exercise, its importance, and its benefits was also quite low, thereby preventing them from maintaining and performing exercise as a part of their routine [13]. From the point of view of the participants in the study, the learning was enhanced by the nature of aerobics as a rhythmically coordinated, high-paced exercise often equated with dance. Consequently, participation and attendance were consistently high throughout the study due to a higher level of interest and curiosity. It was also easier for participants to learn and keep up, as they only had to imitate the instructor and perform the movements to the beats of the music. The music itself, being quite buoyant and loud, also helped in blocking the environmental, emotional, and mental stresses faced by the participant, so more focus and concentration were given to the exercise for a given duration of time. The auditory and visual cues included in this form of exercise also intensified the performance [14]. Tai chi, on the other hand, being an exercise that required a greater level of concentration and attention, was less acceptable to the participants. This form of exercise, being somewhat an entirely new form for them to learn, also made them feel stressed as it was difficult to cope with the new movements taking place one after the other while maintaining a particular stance (wuji stance) throughout the exercise [15]. The limitations of the study include the lack of long-term follow-up and the small sample size.

## Conclusions

This study concludes that aerobics exercises were more effective than both tai chi and control exercises in improving health and maintaining quality of life among the elderly. Aerobics can be recommended as a beneficial mode of exercise for older adults looking to enhance their overall well-being. Additionally, tai chi was found to have clinical benefits, particularly in areas like cognition, static and dynamic balance, cardiopulmonary fitness, and quality of life compared to the control group. This suggests that tai chi can also be a valuable exercise option for older adults seeking to improve specific aspects of their health and fitness.
